# Facile Approaches of Polymeric Face Masks Reuse and Reinforcements for Micro-Aerosol Droplets and Viruses Filtration: A Review

**DOI:** 10.3390/polym12112516

**Published:** 2020-10-28

**Authors:** Yusuf Wibisono, Cut Rifda Fadila, Saiful Saiful, Muhammad Roil Bilad

**Affiliations:** 1 Department of Bioprocess Engineering, Faculty of Agricultural Technology, Brawijaya University, Malang 65141, Indonesia; cutrifda977@gmail.com; 2 Department of Chemistry, Faculty of Mathematics and Natural Sciences, Syiah Kuala University, Banda Aceh 23111, Indonesia; saiful@unsyiah.ac.id; 3 Department of Chemical Engineering, Faculty of Engineering, Universiti Teknologi Petronas, Bandar Seri Iskandar 32610, Malaysia; mroil.bilad@utp.edu.my

**Keywords:** face mask, membrane, aerosol, droplet, Covid-19, virus, filtration

## Abstract

Since the widespread of severe acute respiratory syndrome of coronavirus 2 (SARS-CoV-2) disease, the utilization of face masks has become omnipresent all over the world. Face masks are believed to contribute to an adequate protection against respiratory infections spread through micro-droplets among the infected person to non-infected others. However, due to the very high demands of face masks, especially the N95-type mask typically worn by medical workers, the public faces a shortage of face masks. Many papers have been published recently that focus on developing new and facile techniques to reuse and reinforce commercially available face masks. For instance, the N95 mask uses a polymer-based (membrane) filter inside, and the filter membrane can be replaced if needed. Another polymer sputtering technique by using a simple cotton candy machine could provide a cheap and robust solution for face mask fabrication. This review discuss the novel approaches of face mask reuse and reinforcement specifically by using membrane-based technology. Tuning the polymeric properties of face masks to enhance filterability and virus inactivity is crucial for future investigation.

## 1. Introduction

The demand for face masks during the coronavirus disease 2019 (Covid-19) pandemic keeps increasing. In some countries, medical workers have to deal with a shortage of face masks. Currently, there are many types of masks fabricated from household or cloth material to substitute for a regular disposable surgical mask. So far, only limited studies discuss the filtration efficacy of the custom and the homemade face masks as compared to the regular face masks. The use of face masks made from household material is not recommended for people, especially medical workers [1]. Yet, several research studies have proven that the face masks can be reused after several steps of washing and sterilization [2,3].

Several studies provided data about the filtration efficiency of cloth mask, disposable face masks and N95 respirators for viruses [4,5]. The data show that not even disposable face masks are efficient enough to protect the wearer from getting infected by an influenza-like illness. The utilization of a membrane-based material allows the full retention of viruses through the size exclusion method. However, it requires high-pressure drop, which restricts the wearer’s ability to breath. Nonetheless, this material is seen as a promising alternative. Moreover, some materials have shown great antiviral properties. Hence, this paper reviews recent advancements in polymeric materials and membrane science for the application of face masks with adequate filtration efficiency. The future direction of studies is also briefly proposed to enhance the ability to protect against virus transmission through micro-aerosol droplets carriers.

## 2. Efficacy of Face Masks Against Micro-Droplets and Viruses

### 2.1. Physical Distancing, Face Masks, and Eye Protection

As the pandemic of Covid-19 emerges and the virus spreads easily, several prevention actions have been implemented and regulated to minimize the spread of the virus. The actions include physical distancing as well as wearing a face mask and eye protection, which are believed to effectively prevent the transmission of coronavirus. Physical distancing by self-quarantine is an effective way to stop the spreading of the virus, and yet, as the pandemic lasts for months, the economic deceleration becomes a big concern. People inevitably have to return to work to rebuild and sustain the economic situation. This means that a complete physical distancing would be untenable. Therefore, it is necessary to implement other actions to reduce the spread of the virus such as wearing a mask.

So far, there is no evidence from clinical trials about the effectiveness of wearing a mask or eye protection in preventing the spread of the virus. Nevertheless, washing hands and physical distancing are the prime recommended actions to limit the spreading [6]. The transmission of coronavirus among people spreads from the respiratory droplets through coughing or sneezing. Coronavirus causes the infected people to experience fatigue, dry cough, and fever [7]. Some may not experience any symptoms and only act as a carrier. The asymptomatic carrier is very difficult to be spotted for isolation. This explains why it is very important for everyone to implement every precaution with no exclusion.

The World Health Organization (WHO) has conveyed that wearing a mask is only necessary for people who become ill and those who have taken care of a person with a suspected severe acute respiratory syndrome of coronavirus 2 (SARS-CoV-2) infection. Nonetheless, the recommendations of wearing a face mask in different countries are varied. As awareness increased, people start to take the initiative themselves to wear a mask. For most Asian countries, the governments have made it compulsory to wear a face mask in public areas [8], leading to high demands for the medical face masks and causing the poor supply of masks for the medical workers. The production of the medical face mask and N95 has become greater since then.

Moreover, cloth or fabric-based masks also have also been produced. Hence, several clusters of randomized trials of face masks were done to assess the efficiency of wearing a face mask to minimize the virus spreading [1,9]. The trials concluded that it is not suggested for the medical workers to use a cloth mask when working with people suspected of SARS-CoV-2 infection because it may increase the risk of infection [10]. There are some limitations possess by those masks, in which the cloth mask is claimed to be the most ineffective. The limitations include inadequate filtration efficiency, its poor breathability, washability, and reusability [1,8]. To enhance the applicability of those masks, their limitations can be further studied and overcome.

### 2.2. The Structure of Face Masks

**Figure 1:**
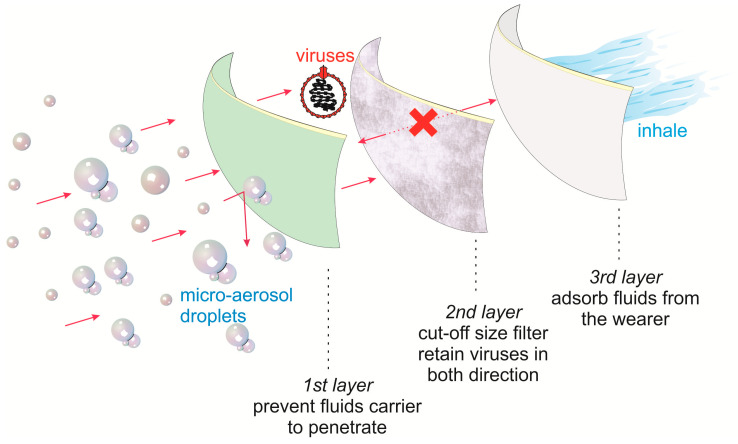
Illustration of the three layers of a surgical face mask to prevent micro-aerosol droplets containing contagious viruses.

Disposable surgical masks are widely used by medical workers, scientists, and societies. Since the emergence of the COVID-19 pandemic, the demand for the masks is increasing as people believed they could protect themselves from the virus infection. SMS (spunbond–meltbond–spunbond) structures are applied for a disposable surgical mask to protect the users from 98% bacteria and to impose hydrophobicity. SMS has the highest level of protection and is the most popular combination structure consisting of 1–5 g m^−2^ melt-blown (MB) microfibers, which have microporous and breathable structures. Surgical masks consist of a very fine middle layer with extra fine glass fibers or synthetic microfibers, which are covered on both sides by an acrylic bonded parallel-laid or wet-laid nonwoven material. The weights of the middle layers are between 10 and 100 g m^−2^, whereas the thicknesses of the fibers are between <1 and ±10 µm. Each layer has a different specific function: the middle layer works as the filter, the outermost layer imposes hydrophobicity, and the innermost layer works as an absorbent to trap droplets coming from the users. The three layers of a surgical mask are expected to work by restricting the transmission of small particles and pathogens from both directions [11]. An illustration of the three layers of the surgical mask is shown in Figure 1.

The medical workers also use N95 to protect themselves, which has a better efficiency compared to other types of masks, including the regular surgical mask. Based on the National Institute of Occupational Safety and Health (NIOSH) 42 CFR Part 84, N95 is actually defined as a respirator, not a mask. The “N” means it is not resistant to oil, and the number “95” indicates that it has 95% filtration efficiencies (FE) to NaCl particles with a particle diameter range from 0.1 to 0.3 µm. The structural components of N95 are composed of the outer layer, the filter layer, and the inner layer. The filter layer is a filter fabric generated from nylon, cotton, polyester, and polypropylene. The fiber diameters of the outer layer and filter layer of N95 are 27.07 µm ± 3.64 µm and 2.79 μm ± 0.95 μm, respectively. The diameter of fibers affects its mechanical filtration characteristics, since smaller fibers form a smaller pore size than bigger fibers of the same thickness. The smaller it is, the higher its mechanical filtration, too. A structural difference is also found between a disposable surgical mask and a N95 respirator. A disposable surgical mask consists of folded piles of fabrics that are loose-fitting on the user’s face, whereas the N95 respirator consists of filtering layers and tight-edge fitting [12].

A global shortage of N95 respirators and regular surgical masks has shifted the demand to household material-based masks that are easier to produce and are now available abundantly. It prompts the study on the efficiency of household material-based masks. The structure materials of this type of mask are usually made of 100% cotton, scarf, cotton mix, or pillowcase. The most efficient household material-based masks based on its filtration efficiency and its pressure drop are 100% cotton masks and pillowcase-based masks. The pressure drop indicates the ability of users to breathe when wearing the mask. A higher pressure drop means that it is harder for the users to breathe. Yet, doubling the cotton layer of the mask does not affect the filtration efficiency significantly but doubles the pressure drop. The study stated that the overall data show that it is possible to partly block viruses or bacteria using this type of mask. However, the performance of these masks is not comparable to the N95 nor to the disposable surgical masks. The advancement of a cloth mask has been reported by combining fabric materials, e.g., cotton–silk, cotton–chiffon, and cotton–flannel [8]. Such combinations increased the filtration efficiency to 80% (<300 nm particles) and 90% (>300 nm particles) [8].

### 2.3. Materials for Face Masks Manufacturing

A surgical mask can be produced by using woven, non-woven, and knitted methods. However, non-woven is the most common method, and it costs less. Surgical face masks were manufactured by using 20 g m^−2^ polypropylene with spunbond technology, whereas it needs 25 g m^−2^ non-woven sheet polypropylene using melt-blown technology. Polystyrene, polyethylene, polyester, and polycarbonate could also be used to manufacture the surgical face masks. The filtration efficiency of the surgical mask is influenced by several factors such as its fiber selection, fabrication method, the structure of the web, and the cross-sectional shape of the fiber. Surgical face masks can be classified into three categories, which are a low barrier, moderate barrier, and high barrier. A moderate barrier type of mask has a Bacterial Filtration Efficiency (BFE) of ≥98%, whereas a low barrier has a BFE ≥ 95% [13].

Generally, a disposable surgical mask consists of three different layers made of non-woven fabric. They are cheap and easy to fabricate. The fabrication of non-woven fabric takes place by using a polymer as the material treated with heat, chemical, and mechanical means. Generally, the fabrication occurs based on the spunbond method or melt-blown method. The spunbond method begins with the extrusion process where the polymer is melted by the heat and mechanical action. It is necessary to maintain the required temperature to melt the polymer. The molten polymer is shaped into thin filaments by a spin pack. Subsequently, the thin filaments were quenched by cool air and bonded together by heat, chemical, or mechanical means to form the non-woven fabric. Although the spunbond and melt-blown methods are similar, there is one significant process that differentiates them. The die process in the melt-blown method is very important and responsible for the formation of the microfibers pore size. The die process consists of feed distribution to ensure the molten polymer spreads evenly, the die nosepiece to ensure filament diameter and quality, and the air manifold where the polymer filaments become much thinner microfibers. Moreover, the meltdown method generates much finer microfibers and creates a smaller pore size. Hence, it is usually used to produce the filter layer in the disposable surgical mask [8]. The N95 respirator also comprises of three polypropylene fiber layers: outer (two layers), filter (one layer), and inner (seven to eight layers). The outer and the inner layers are fabricated with the spunbond method, whereas the filter layer is fabricated with the melt-blown method. The typical thickness of the outer, filter, and inner layers are 300–400, 800–1200 and 100–150 μm, respectively [14].

### 2.4. Disposable Surgical Mask Versus N95 Health Worker Mask

It is recommended that the medical workers use the N95 instead of the disposable surgical masks. Recent studies assessed the efficiency of both the N95 and the disposable surgical mask using actual data. A research study was conducted to assess the efficiency of a N95 respirator and a disposable surgical mask by comparing the values of their Particle Filtration Efficiency, BFE, Viral Filtration Efficiency, and the NIOSH NaCl Efficiency [4]. Significant differences in the results between the surgical face mask and the N95 are shown by the assessment with NIOSH NaCl Efficiency, in which the value for the surgical face is lower than the N95 (as shown in Table 1). The NIOSH NaCl Efficiency method can identify a poor filtration performance of the masks and is commonly used for the certification process of a filtration device by assessing particulate-filtering and air-purifying respirators’ efficiency. The filtration tests were done using an Automated Filter Tester with aerosolized NaCl solution sizes that ranged from 0.022 to 0.259 μm. NIOSH certification tests usually use 90–100 min of penetration to load 200 mg of aerosolized NaCl [4].

In addition, efficiency test results were also obtained with randomized control trials to assess the performance of the N95 and the disposable surgical masks. A randomized control trial involves participants that were using disposable surgical masks and N95 respirators in the hospital (handling patients with a respiratory infection). The research was done by utilizing meta-analysis from 29 previous studies and six clinical studies and showed that the medical workers that were using N95 respirators and the ones with surgical face masks have an insignificantly different risk of getting a respiratory infection. Although randomized control trials give an actual clinical situation of results assessment, this type of research comes with several risks of bias; for instance, the research did not measure the hand hygiene of the medical workers [1,9].

### 2.5. The Limitation of Commercially Available Face Masks

Randomized control trials and laboratory tests show that the current cloth-based face masks still pose some limitations [1,15]. Such a claim was based on laboratory testing of filtration performance with a TSI 8110 filter tester by a known concentration of sodium chloride particles of a specified size range and at a defined flow rate. The penetration of particles on a cloth mask shows a very high percentage compared to other types of medical face masks, as shown in Table 1 [15]. Furthermore, the risk of getting infected might originate from self-contamination because of repeated uses of the cloth mask. According to another study, after the 4th washing and drying of the cloth masks, the pore size enlarges, which drops the filtration efficiency by 20%. The deflation of its efficiency is also affected by the lack of microfibers within the pore region. However, despite the limitations, there is still the potential to develop cloth masks by choosing the proper material and layers of the cloth. It is possible to develop a cloth mask by exploiting the number of layers, layer density, and facial fitness, and yet at the same time still considering the breathability, washability, and reusability [16].

A disposable surgical mask requires a minimum of 80% filtration efficiency to protect the users from bacteria. It is not designed to be used more than once. It protects the users by reducing the risk of getting any body fluid such as blood, but it is not adequate protection to prevent the users from getting infected by a respiratory infection such as Covid-19. On the other hand, a respirator such as N95 is a device that reduced the exposure of the user’s respiratory to airborne contaminants or particles that are small enough to be inhaled by humans. Although the N95 respirator appears to be better protection compared to other types of masks, the efficiency in blocking 300 nm particles is just around 85%, due to its wider pores (pore sizes of N95 ≈ 300 nm), and the Covid-19 viruses have particle sizes around 65–125 nm [4]. Hence, it is still necessary to further develop this type of mask to increase its efficiency in protecting the users from getting infected by the Covid-19 virus.

## 3. Membrane Technology for Virus Filtration

### 3.1. The Basic Principles of Membrane for Air Virus Filtration

Generally, the process of air filtration can be done either with membrane or depth filters. Depth filters work by retaining the particles on the filters, whereas the membrane retains the particles on its surface. A membrane typically composes of a thin porous polymeric film with specific pore sizes. The purification of air from viral particles happens as the viruses are retained on the surface of the membrane due to its smaller pores.

**Figure 2:**
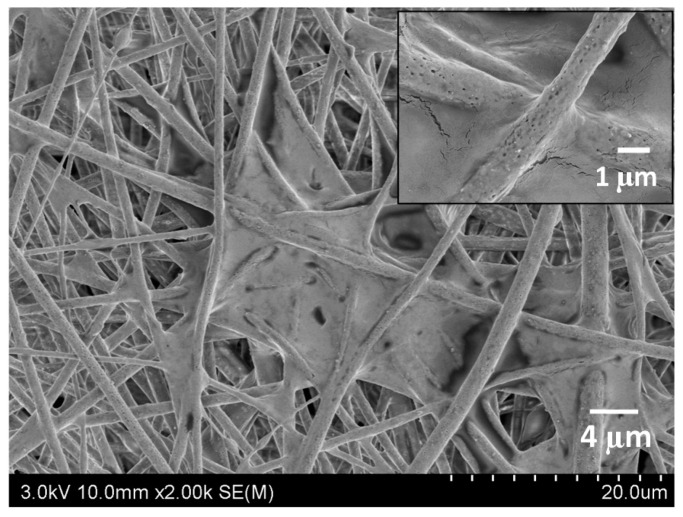
PLA/chitosan fibrous membrane for air purifier [19].

The membrane-based materials have been used for the application of virus filtration. Membrane material has a great potential to filter viruses, as the pore size of the polymeric membrane filter can be adjusted according to its application. In fact, this technology has become a standard for the separation of viruses on biopharmacy thanks to its high filtration efficiency. Typically, virus filters are composite membranes that provide both mechanical properties and virus retention [17,18]. The virus filtration has been successfully done to filtrate retroviruses (>50 nm) and parvoviruses (18–24 nm). It shows excellent virus retention, which led to very high market demands. Unlike for virus filtration in aqueous media, the airborne virus filtration technologies have been used for personal respiratory protection or in air-purifiers. A poly (lactic-acid)/chitosan fibrous membrane, as shown in Figure 2, was used to filter an artificial polluted environment with a removal efficiency of 100% in 33 min [19]. However, this filtration technology also comes with a drawback where a cake formation on the surface of a membrane occurs after several time of prolonged usages [17].

The efficiency of filtration with membrane technology is largely dependent on its pore sizes and distribution. The airflow rate will not affect the separation efficiency through a membrane. However, it is also considered as an important factor due to its relation with the breathability of a mask. According to the NIOSHs, a mask’s airflow rate needs to be more than 85 L/min. As presented in previous research, a higher airflow rate will decrease the filtration efficiency of a mask. Yet, the airflow rate can still be increased by increasing the overall material porosity without decreasing the filtration efficiency. The main challenge of using a membrane for virus filtration is how to reduce and clean the foulant materials [20].

### 3.2. Materials of Membrane for Virus Filtration

Almost all bacteria and viruses can be removed by ultrafiltration membranes, which usually requires a low transmembrane pressure and is lower in cost [21]. Successful virus filtration is also affected by the material of the membrane. Positively charged polymers have been proved to possess a resistance toward the virus [22]. It can damage the lipid membrane of the enveloped viruses and capsids of non-enveloped waterborne viruses. A polyethyleneimine (PEI) is a polymer material that gives antimicrobial and antiviral properties and is known as an efficient transfection agent with low cytotoxicity [16,22]. Based on previous research, a modified membrane with PEI-coated polyethersulfone (PES) materials have induced a tremendous ability to reduce viral presence by 99.9%, which was never reached before in other research studies [22].

Cationic polymer materials interact with the virus’s cell membrane and destroy it after successfully permeating the cell walls. The destruction of the cell wall occurs due to interaction between the amino groups of PEI and cell membranes. It is reported to have optimal properties at molecular weights of 9, 10, and 24 kDa. The cationic polymer was used in previous research as an anti-HIV-1 material in which the filtration efficiency was increased as the molecular weight of the PEI increases [23]. Moreover, zwitterionic hydrogen was also found to be effective for virus removal in water reuse, as it creates a repulsive interaction between the virus and the membrane. The interaction overcomes the fouling problem on the PES membrane surface because it prevents the virus from approaching the membrane surface, thanks to the modification with a grafter layer of poly [3(methacryloylamino) propyl] dimethyl (3-sulfopropyl) ammonium hydroxide hydrogel. Hence, it weakened the accumulation of the virus on the membrane’s surface and eventually increased its filtration efficiency [24]. Moreover, surface patterning of the membrane has also been proven to be an effective technique to overcome the fouling problem. It prevents the deposition of particles on membrane’s surface, which can be induced by a template micromolding method or direct printing method [25].

The application of polyphenols onto masks was also proved to be effective for antiviral properties. Polyphenols are secondary metabolites that can be extracted from many kinds of plants. The antiviral properties of polyphenol are due to the existence of catechin and theaflavin molecules that bond with the virus’s nucleic acid. Catechin with 98% purities was used via grafting it on commercial non-woven cellulose layers. The modified cellulose layers successfully enhanced the antiviral properties of commercial masks. However, the use of three modified layers decreased airflow, which makes it less breathable [26].

### 3.3. Reinforcing Face Masks by Using Membrane Filters

The development of face masks has been studied to increase the filtration efficiency of face masks and to add other functionalities. A lot of aspects can still be improved. The efficiency of prime protection such as N95 respirators is still only at 85% efficiency because of the pore size of 300 nm, which is much larger than the size of the Covid-19 viruses of around 65–125 nm [20]. Therefore, several studies were conducted by applying membrane technology to reinforce the currently established face masks. This finding suggests that it is still possible to enhance the filtration efficiency of face masks using a membrane filter with a specific pore size to almost completely prevent the virus from passing through the mask’s filters [27].

Three established parameters to assess the overall filtration performance of masks are the filtration efficiency, breathability, and durability. The implementation of membrane technology is intended to not only increase filtration efficiency but also to increase filtration efficiency and provide a comfortable face mask. Recently, membrane filters with enhanced characteristics such as smaller pore size, lighter weight, low air resistance, and a more active surface membrane by electrostatic forces or chemical bond interaction have been discovered. Particulate matters removal efficiency can be increased by using membrane filters with nanosized diameter fibers, which are also called a nano fiber membrane [27].

The fabrication of a nanofiber membrane can be conducted using the electrospinning method, and polymer as the main material yields to a great filtration efficiency (>95%) with an average fiber diameter of ≈200 nm, sufficient pressure drop (132 Pa), and lightweight. Another newly developed interconnected membrane shows an even higher filtration efficiency of 300 nm aerosol particles: up to 99.99%. Moreover, it yields a tremendous quality factor (0.1163 Pa^−1^) under a high flow rate (90 L min^−1^), which is significantly better than regular masks [8]. Electrospinning technology can generate a uniform diameter nanofiber with a given diameter where the filtration efficiency will decrease as the nanofiber diameter increases. The nanofiber’s utilization is a significant part of reinforcing regular face masks, as it shows excellent characteristics such as high permeability, low basis weight, and small pores. Nanofiber filter media could retain particles with nanosized pores ≈ 100 nm with very high virus retention efficiency and simultaneously offer adequate breathability due to the ability to reduce airflow resistance on masks. It has been proven in previous research that the incorporation of nanofibers onto disposable surgical face masks has shown greater filter efficiency [27]. Typical commercial surgical masks have an efficiency between 80.57% and 84.78% [27].

Many recent developed electrospun air filters show better properties. For example, the composite air filter membranes generated by electrospinning a mixture of polyvinyl chloride and polyurethane polymer (PVC/PU) demonstrates good mechanical properties with a tensile strength up to 9.9 MPa with an excellent air permeability (706.84 mm s^−1^), a high filtration efficiency (99.5%), and a low pressure drop (144 Pa). A composite membrane fabricated with Nylon 6 and PAN was found to be showing an even higher filtration efficiency (99.99%). Moreover, the superhydrophobicity and superoleophobicity of the electrospun nanofiber composite membranes can be adjusted. Another developed air filter of a multilayer structured air filter membrane has been studied. Wan et al. conducted a superhydrophobic eletrospun air filtration medium after they spun the PSU with TiO_2_ nanoparticles and deposited it onto a conventional non-woven substrate. This fabrication of layers also achieved very high filtration efficiency (99.9997%) with a pressure drop of 45.4 Pa. A multilayer structured air filter membrane with a high filtration efficiency of 99.989% (for 300–500 nm NaCl aerosols) was also conducted by using PAN nanofibers with incorporated silica nanoparticles [28]

Despite of the all advantages of the general electrospinning method, it comes with a great environmental impact. Hence, it should be highlighted that it is important to imply the green electrospinning for the fabrication of a nanofiber membrane. For example, the materials used in the electrospinning process can be substituted with natural polymers such as polysaccharides, proteinaceous materials, and natural waste, biosynthetic polymer materials, and the chemical synthesis of polymers. In addition, the use of harmful solvent can be altered with water as the electrospinning solution where a water-soluble polymer is used such as polyvinyl alcohol (PVA), polyethylene oxide (PEO), polyamic acid (PAA), and hydroxypropyl cellulosecan (HPC) [29].

Polyamide nanofiber was successfully obtained by electrospinning the ammonium salts of PAA via aqueous solution. An eco-friendly electrospun membrane was also conducted by Lv et al., where the membrane was fabricated by using polyvinyl alcohol (PVA), konjac glucomannan (KGM), and ZnO nanoparticles via green electrospinning and eco-friendly thermal cross-linking. The ZnO nanoparticles enhanced the filtration efficiency up to 99.99% and increased the pressure drop to 130 Pa (300 nm particles). Moreover, the solvent-free electrospinning methods have also been studied (i.e., melt electrospinning, anion-curing electrospinning, UV-curing electrospinning, thermo-curing electrospinning, supercritical CO_2_-assisted electrospinning) as efficient and environmentally friendly manufacturing processes [29,30].

### 3.4. Face Mask Reuse

Due to a shortage of face masks during the corona pandemic, different types of face masks were fabricated with option for reuse. This led to some studies on the reusability of face masks. A disposable surgical face mask can be reused after a proper sterilization. A proper sterilization can be done using a dry sterilization process or regular steam process for 15 min at 121 °C where the face masks were placed in sealed bags [3]. These processes are considered as an efficient way to sterilize face masks by inactivating the coronavirus. Previous research assessed the effectiveness of this method through blind comparison of a used mask with an unused mask of permeability properties, pressure drop, and filtration capacity. The results show that no differences were found between the used and unused mask, even after multiple sterilizations. Hence, dry sterilization and a regular steam process can be a great option to deal with the shortage of masks in hospitals [3].

As mentioned before, fibrous filters have shown many great advantages as materials for air filters. They have been developed by adhering antimicrobial components onto filter fibers such as silver, cobalt, and titanium oxide nanoparticles. However, the incorporation of nanoparticles on filters will decrease its durability for reuse. This problem was solved by fabricating a fibrous filter with non-woven polyethylene terephthalate (PET), which was treated with titanium isopropoxide and dip-coated into AlH_3_{(OC_4_H_9_)_2_}. Although PET/Al filters were fabricated using a chemical process, they did not show any significant change in the filtration performance. The coating of the PET filter with Al improved its durability, which was shown from its stable performance even after washing the filters multiple times [2].

## 4. Integration of Membrane Technology and Other Technology for Virus Filtration

### 4.1. Electrocharge Polymer Fabric

Filters with electrocharge can retain the particulates by an electrostatic interaction rather than size exclusion. Positively charged filters are needed to filter viruses, which usually have a negative charge. Recent studies have employed electropositive filters for virus filtration in water. In contrast, electronegative filters have been used and mixed with cellulose ester membrane with nanosized pores between 450 and 100 nm and effectively retained enteroviruses. Although viral particles have smaller sizes than the pore sizes of a membrane, the retention of viruses still happens due to the presence of salts (cation valence), which yielded to virus adsorption. Moreover, the increase of pH value (acidity) will also affect its efficiency to adsorb viruses. The results of previous research show that at pH 7, the presence of salts increased virus adsorption. On acidic conditions (pH 3.5), more than 95% of viruses were adsorbed [31].

Filtering face piece respirators comprise composite structures with multiple layers where a central filter layer consists of electret properties. The central layer can be made by synthetic polymer fibers, e.g., polypropylene, polybutylene terephthalate, and polytetrafluoroethylene. Electrostatic charging was delivered into the central layer by corona discharge, triboelectrification, or electrostatic spinning. Furthermore, particles were retained effectively because of mechanical action and electrostatic forces. In contrast, the filter layer fabricated through the melt-blown method will not possess the electret properties. Hence, the filtration process will only happen mechanically, which decreased its virus retention efficiency [31].

### 4.2. Si-Based Nanoporous Template

**Figure 3:**
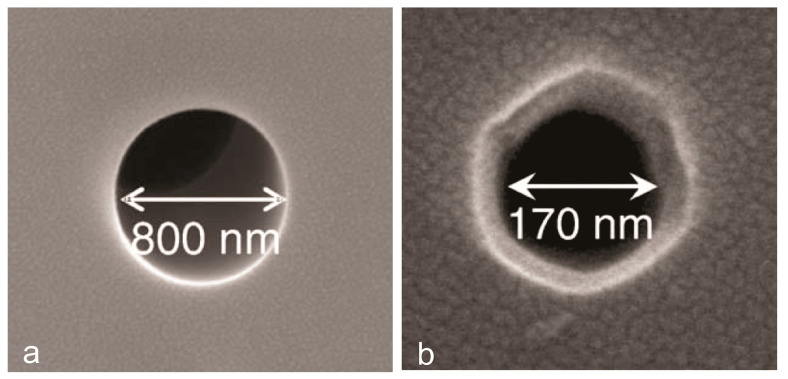
Formed pore from 90 nm silicon nanowire template etched with XeF_2_: (**a**) 200 cycles and (**b**) 50 cycles etching process [33], reproduced with permission. Copyright Elsevier Ltd.

Since 2007, an Si-based nanoporous template has been developed with its ability to form pores spontaneously during the crystallization of silicon film. It is capable to form pores of ≈5 nm with a very narrow pore size distribution [32]. Silicon nanowire templates can be produced by the chemical vapor deposition (CVD) method. CVD involves the deposition of gold nanoparticles on a silicon substrate. A silicon precursor (SiCl_4_) was added into the CVD reactor containing a silicon substrate at 850 °C temperature where the silicon nanowires were subsequently formed vertically. Then, the polyimide membrane was fabricated after adding the polyimide solution onto silicon substrate nanowires, which were etched with O_2_ plasma, and KI/I_2_ solution. This process involves XeF_2_ as an etchant to remove the silicon component of nanowires. The results show that the membrane’s pore sizes were influenced by the template diameter and duration of XeF_2_ etching, whereas the thickness of membranes depends on the amount of poured polyimide solution. This research was successfully conducted on membranes with pore sizes dependent on diameter templates ranging from 800 to 170 nm [33]. The smallest pore was found by applying 50 cycles of etching using a diameter template of 90 nm (as shown in Figure 3).

The Si-based nanoporous template has been widely used thanks to its versatility to fabricate a membrane. It generates a membrane with an array of pores in a size range between 500 nm and 5 μm, which are commonly used in electroosmotic pumps. According to El-Atab et al., [20], a higher KOH etch rate on silicon releases a microneedle array, which has been studied for use in DNA separation. Membranes are formed by the potassium hydroxide etching process of the macroporous silicon wafers backside. The etching process takes place by utilizing the formation of a coupled chemical–electrochemical system by KOH and Si [34]. The etching process determines the pore size and shape of a membrane where an exposed part will be transformed into a hollow channel [35]. Conventionally, the etching process involves 25% KOH at 80 °C with a rate 1.3 μm min^−1^ or at 90 °C with 2.8 μm min^−1^ rate. Potassium hydroxide did not enter into the pores or dissolve in the membrane, because it was already oxidized thermally with silicon dioxide, which was forming a thin etch stop layer. The thin etch layers will subsequently be removed using hydrofluoric acid to complete the fabrication of the membrane [34].

The fabrication of a silicon-based template involves two main processes: patterning and KOH etching to produce a material with a nanosize range of pores. Silicon-based patterns were generated by exposing the SOI wafer coated by a SiO_2_ hard mask to e-beam lithography, where an array of 90 nm by 90 nm was obtained with a spacing adjusted to be 200 nm. The process was continued with the Reactive Ion Etching (RIE) system to remove the exposed area of a SiO_2_ hard mask. Subsequently, the etching process with KOH was done to achieve V-grooves. The etching time with KOH 44% affected the nano-aperture sizes, which was increased as a function of time. The created template can be used to fabricate a membrane with polyamide as a hydrophobic material. A SiO_2_ hard mask was used to utilize the lower energy of Si than PI, which facilitated the membrane being peeled off easily after being etched. Polyamide film would be etched with the RIE system to create cavities with a nanosize range on the film [20].

### 4.3. Hydrophobic Flexible Membrane

**Figure 4:**
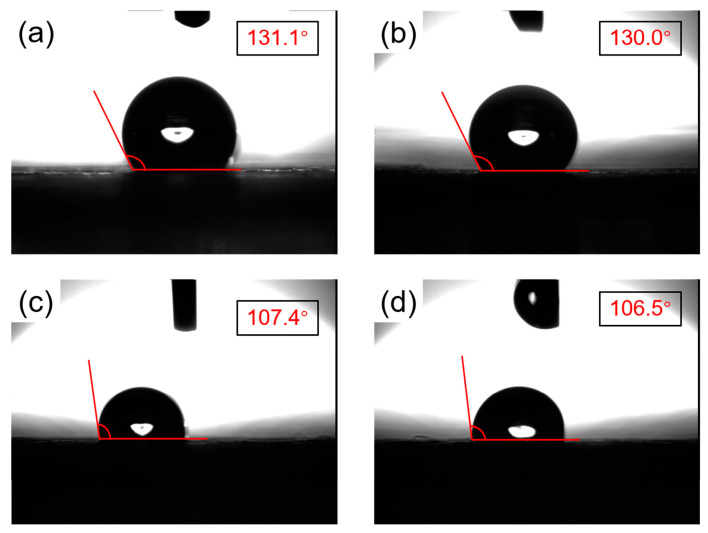
Optical contact angle measurements of PTFE membranes for air filtration: (**a**,**b**) nanofiber, (**c**,**d**) microfiber [39].

There are two types of surface membrane properties, either hydrophobic or hydrophilic. The hydrophobic membrane is known as “water repellent” as it is a prohibiting surface to get wet. Water droplets will be formed on the surface of the hydrophobic membrane due to its low surface energy. Hence, a membrane’s hydrophobic properties are a great advantage in its application to develop face masks [36]. Nowadays, electrospinning has been a widely used technology to fabricate a flexible and permeable membrane [37]. A polymer membrane with a very high contact angle (>150°) was conducted from previous research by using PVDF/ZnO nonofiber fabricated with electrospinning technology. A very high contact angle (>90°) indicates the great hydrophobicity properties of a membrane. Its high hydrophobicity gives a self-cleaning surface where the particles that were retained on the surface will be washed away with water droplets, leaving a dry and clean membrane [38]. The feasibility of polytetrafluoroethylene/poly (vinyl alcohol) PTFE/PVA nanofibers electrospun on a PTFE microfiber membrane was investigated. The composite membrane was a hydrophobic membrane (as shown by optical contact angle measurements in Figure 4), which demonstrated an increase of filtration efficiency to 98.905% and 100% for PM2.5 and PM7.25, respectively [39].

There are several factors affecting membrane surface wettability such as the surface energy, surface roughness, and surface tension of a liquid. Surface energy is induced due to the relationship between the cohesion force and the adhesion force. In addition, liquid surface tension also influences membrane wettability where stronger intermolecular forces raise surface tension. When a liquid droplet is placed on the membrane surface, it will form a droplet contact angle of more than 90°, which is indicating its hydrophobicity. Furthermore, a super hydrophobicity material has already been developed with a contact angle of more than 150°. The materials used to fabricate hydrophobic membranes are usually polymers with low surface energy such as polyethylene (PE), polypropylene (PP), PET, and polyvinylidene chloride (PVDC). A development of hydrophobic membranes with hydrophilic monomers such as AA, HEM, As, Ct, and Ma with OH functional groups has been discovered. The hydrophilic monomer’s addition yields a formed nanopore size top layer of membrane-enhanced surface roughness and hydrophilicity [36].

According to previous research, a silicon-based nanoporous template generates a hydrophobic membrane film that is effective in reducing the membrane fouling. A filter with a hydrophobic surface is favored because it repels droplets to slide over the face mask instead of being retained on the surface, forming a cake. This method shows good results, as demonstrated by the SEM images. It can generate a polyamide membrane with a narrow distribution of pore sizes, which vary between 5 nm and 55 nm depending on the etching time with KOH. Moreover, the pressure drop of the membrane was also calculated to make sure it still has an adequate breathability, as it will be attached to an N95 respirator. The calculation results show that wider pores give a higher airflow velocity (higher breathability) but reduce filtration efficiency. The spacing also affects airflow velocity. It is necessary to have ≈300 nm spacing to achieve a standard airflow velocity (of >85 L min^−1^), which increases at higher pore sizes. Yet, it is possible to overcome this problem by applying several patterning steps to increase the porosity and breathability of the membrane [20]. 

Since membrane filtration works as it strains particles on the surface, cake formation will also be an issue. A formed cake on the membrane’s surface will block pores and further reduce the airflow velocity. However, due to the hydrophobic characteristic of polyamide and a large inclination angle of the membrane when it is attached to a mask, it is supposed to have a self-cleaning system. The droplets will be rolled over the polyamide film. Therefore, it will not reach the wearers, as it might carry coronavirus [20].

### 4.4. Electret Polyethersulfone/Barium Titanate Nanofibrous Membrane

Airborne pollution and pathogens have been an issue for human health and have become a great concern, leading to a highly desired face mask with adequate performance to protect the wearer from getting exposed to contaminated air. A study about an electret polyethersulfone/barium titanate nanofibrous membrane was conducted by integrating it on a non-woven polypropylene. A disposable surgical mask and respirator composed of polypropylene non-woven fabric were produced by using the melt-blowing method. The melt-blown layer’s polypropylene has a diameter between 1 and 10 µm with a thickness around 100–1000 µm, which is not an adequate characteristic to have a great performance to filter fine particles such as viruses that have nanosized particles [38]. Other fibrous materials such as glass fibers and spunbonded fibers are also inadequate to perform the filtering process due to its high pore size. Hence, to increase filtration efficiency, some methods have been applied such as corona charging, thermal poling, and triboelectrification. Those methods are considered as promising in obtaining an efficient filtration process. A long-range electrostatic force and the creation of countless electrodes on the periphery fibers have improved the filtration efficiency. However, a major concern was found, in which they cause a serious limitation of airflow due to its high basis weight and low porosity, which is associated with the breathability of wearers, moisture, and thermal radiation [40].

The electrospinning process is considered as an advanced technology to fabricate an ultralight nanofiber membrane because it results in pore morphology with high tortuousity, a tunable pore size and shape, and good packing density. Hence, it is a promising technology to fabricate a filter material with a high filtration efficiency with its ability to capture particles. Moreover, barium titanate (BaTiO_3_) can be used as the main material for the ferroelectric inorganic electret in the electrospinning process and to fabricate the desired properties of a nanofiber membrane. In addition, polyethersulfone (PES) was selected as a precursor polymer as it has excellent properties, indicating it as an excellent material for an air filters. It resulted in a composite breathable nano-membrane, which also provides the radiative cooling properties suitable for a mask [40].

The fabrication of the nanofibrous membrane used barium titanate (BaTiO_3_, particle size 20–60 nm), polyethersulfone (PES), N,N-dimethyformamide (DMF), and N-methyl-2-pyrrolidone (NMP). The dope solutions were prepared using DMF/NMP solvent with a varied weight ratio and the mixture solution of PES and various amounts of BaTiO_3_. The results show that as the ratio of DMF/NMP decreased, the fiber diameter was also decreased down to 115 nm. The deflation of the formed fiber’s diameter might be influenced by a high boiling point of NMP. The data also show that a parameter of air filtration is significantly affected by solvent properties. Based on the characterization with NCl aerosol particles, a higher solvent concentration decreased both the filtration efficiency and pressure drop of the resulting nanofiber membrane. Moreover, the addition of an inorganic electret greatly enhanced the filtration efficiency, as its presence decreased the fiber diameter of the membrane with a more homogeneous size as a result of the prompt evaporation of solvents during the separation phase. A higher value of softness of the membrane was also achieved by injecting more BaTiO_3_. An injection of 2% BaTiO_3_ led to the formation of the smallest pore size of 1.47 μm with a maximum pore size of 2.56 μm. It also resulted in a better moisture transferability related to the comfort for the mask’s wearers. Moreover, a composite membrane consisted of PES and 1.5% BaTiO_3_ showed a great filtration efficiency (99.99%), low pressure drop (67 Pa), and low basis weight (4.32 g m^−2^) [40].

### 4.5. Colloidal Silver Nanoparticle-Reinforced Membrane

A protective layer with great hydrophobicity and dryness is a very important attribute of face masks. A moist environment will lead to the growth of bacteria, which will cause a health risk for the wearers. Nowadays, the utilization of nanoparticles (e.g., titanium oxide, zinc oxide, copper, gold, magnesium, chitosan, silver, and alginate) has been widely explored to impose antimicrobial properties on masks. Commonly, due to their unique optical, physicochemical, and biological properties, silver (Ag) nanoparticles have been used in various fields [41]. It has been proven that the incorporation of nanoparticles (gold) was successfully conducted to form a membrane with a very narrow pore size distribution, which is an important factor in generating a filter layer for viruses [17].

**Figure 5:**
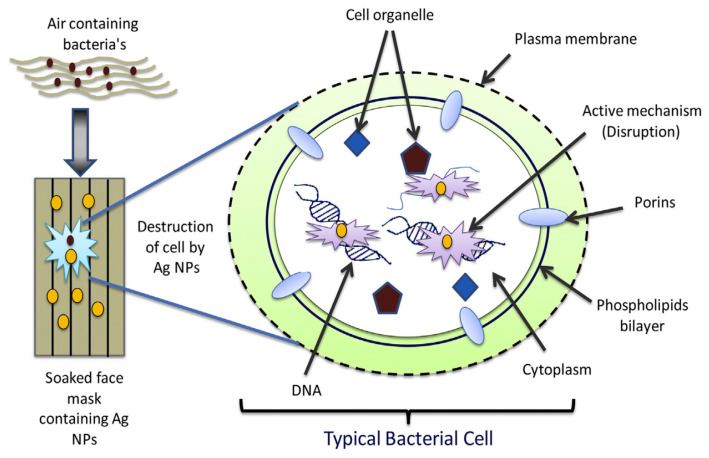
Antimicrobial effects after incorporation with silver nanoparticles [41], reproduced with permission. Copyright Elsevier Ltd.

Silver nanoparticles possess great antimicrobial and antifungal properties. Basically, the nanoparticles are incorporated into the material of a mask, which can be done by using several different methods such as sol–gel, photochemical, electrochemical, sonoelectrochemical, light-assisted, the silver salt method, etc. The silver salt method is considered an easy synthesis procedure, as it requires less time for the synthesis and the formation of high-purity silver nanoparticles. Moreover, the addition of reducing agents such as dimethylformamide, polyvinylpyrrolidone, etc, result in silver nanoparticles in a range size between 1 and 10 nm. However, the use of reducing agents has been replaced with more eco-friendly agents, such as starch to replace the toxic chemical reducing agents [41]. The mechanism of antimicrobial properties is illustrated in Figure 5.

In a previous study, silver nanoparticles were formed in the surface of a face mask to improve its resistance to bacteria growth. The incorporation of nanoparticles was done by soaking the face mask in a silver colloidal solution prepared using starch, AgNO_3_, and N_2_H_4_. Silver nanoparticles were successfully incorporated onto the face mask as proven from characterization of the treated mask with the UV-visible spectroscopy and the EDAX analysis. The mask with silver nanoparticles shows that it effectively inhibits bacterial growth *(E. coli* and *S. aureus*) by destroying the bacteria’s cells via DNA. The findings demonstrate the efficacy of silver nanoparticles for biomedical application [41].

Silver nanoparticles have also been used in membrane technology as they show great advantages. The silver nanoparticles are typically incorporated onto the membrane. The general fouling in the membrane can be overcome by adding silver nanoparticles, which will lead to better permeability, selectivity, structure robustness, anti-fouling, antimicrobial, and photodegradation properties [42]. A previous study compared triangular silver nanoparticles and spherical shape silver nanoparticles regarding antifouling polymer-membrane performance. A polyethersulfone microfiltration membrane with a pore size of 0.1 µm was modified with silver nanoparticles ≈30 nm. The results show that the modified membrane with triangular-shaped silver nanoparticles has improved antibacterial killing efficiency (100%), which is better than that of the spherical-shaped silver nanoparticles (91%). Higher efficiency was achieved because of the high atom density active facets of triangle silver nanoparticles, where its sharp tip increased the negative charge on the membrane, resulting in an improved anti-adhesion and reduced biofouling on the membrane. The modified membrane also poses an enhanced flux of 36% and high flux recovery of up to 96%. Moreover, the modification with silver nanoparticles with a triangle shape gives long-term membrane stability for up to 4 months of usage [43].

According to another research study, silver nanoparticles have been proven to have great resistance not only to bacteria but also viruses. Similar to bacteria, these nanoparticles exert an antiviral activity by penetrating the cell membrane of viruses and interacting with the viral genome. The interaction between virus cells and nanoparticles prevents the cell from replicating. The utilization of nanoparticles has been studied to analyze the effectiveness of its capability to give inhibitory effects toward viruses such as influenza virus, monkey-pox virus, HIV, hepatitis B virus, etc. [44].

### 4.6. Future Perspectives

**Figure 6:**
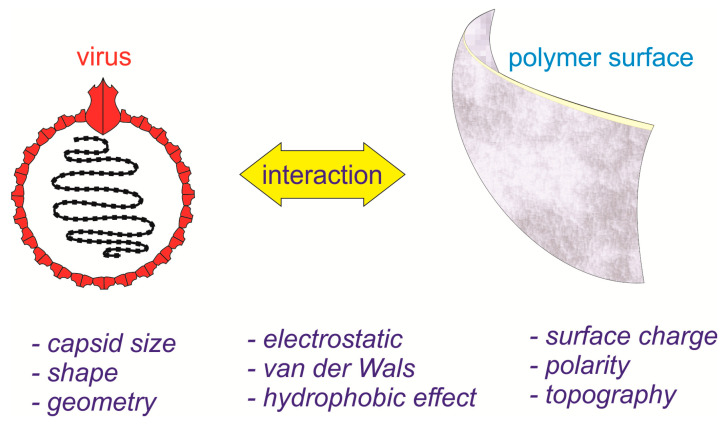
Tuning polymer-specific properties to enhance filtration efficiency and virus ineffectuality.

Apart from those discussed earlier, the interaction between viruses and the filter surfaces is important. Virus adsorption onto surfaces was affected by electrostatic interaction, hydrophobic effect, van der Wals interaction, hydrogen bonding, and steric hindrance. The interaction depends on the capsid size, shape, geometry of the virus, and the surface charge, polarity, and topography of the surface [45]. In liquid filtration, the interaction might be affected by pH and salts, which influenced the hydrophobic interaction of viruses and the surfaces [46]. Yet, in airborne viruses, the aerosol droplet carrier size affects the infectivity and survivability of viruses [47,48]. In order to enhance the filtration efficiency and virus ineffectuality of face masks, tuning the polymer-specific properties which are capable of capturing micro-aerosol droplets and adsorbing the coronavirus become critical (schematic overview shown in Figure 6).

**Figure 7:**
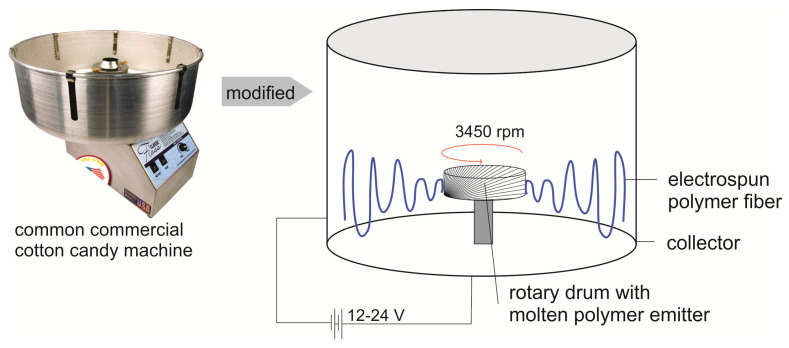
A commercially available cotton candy machine can be slightly modified to produce polymeric electrospun fibrous membranes for face masks in a very tough situation, adapted from [50]. PET bottles could be used as polymer materials.

Furthermore, a newly developed method has been discovered for the fabrication of fibers, namely, force spinning technology. Through this technology, fibers with a size range between 100 and 600 nm can be obtained. The working principle of the force spinning technology is similar to that of a cotton candy machine, where the polymer is melted from the center and ejected to orifices. Subsequently, fine webs of fibers are obtained after the continuous stretching of the melted polymer and finally collected on the collector system. Several factors affect the formation and morphology of the fibers. They include the solution concentration, viscosity of the melted polymer, rotational speed, the distance between collection system and spinneret, and the gauge size of the spinneret. This technology comes with several drawbacks: it needs a large-scale production, continuous fiber collection on roll goods, and extension to spin a variety of polymers in addition to polypropylene and nylon [49]. However, this technology is simple, by slightly modifying a common cotton candy machine (as shown in Figure 7), and it demonstrated the ability to produce electrocharged polymer materials such as polypropylene. During the very difficult global situation at present, the use of polystyrene or polyethylene from plastic bottles, for instance, is possible [50].

When the emergency situation in providing face masks passes, the development of biodegradable face masks is essential. The use of non-biodegradable polymer face masks was expected to produce 66,000 tons plastic waste in UK, and the number will be significantly higher for the total world population. The production of biodegradable face masks made from natural biomass or green polymers is a must for providing an environmentally benign solution. Based on a Life Cycle Impact Assessment (LCIA) study, the use of reusable or biodegradable face masks will lead to a 95% reduction of waste [51].

**Figure 8:**
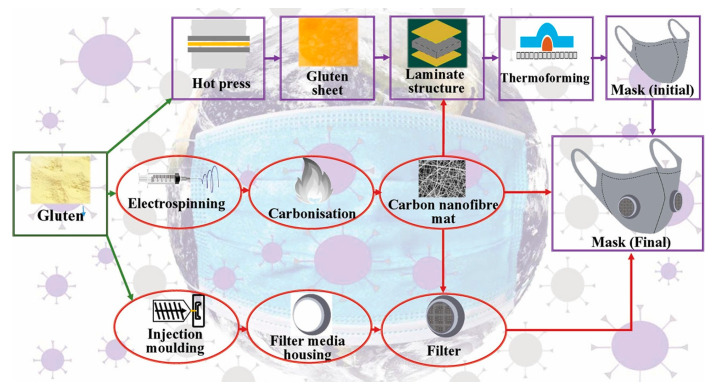
Proposed pathway to produce gluten-based and biodegradable face masks [52], reproduced with permission. Copyright Elsevier Ltd.

As a co-product or by-product of cereal industries, biopolymer from wheat gluten can be made for gluten-based face masks by electrospinning to produce nanofiber membranes and the hot press method to produce gluten sheets, as shown schematically in Figure 8 [52]. Other nature biopolymers from wood fibers such as pine, cedar, spruce, and other softwoods were under development to use as a biodegradable face mask as those fibers could be easily found in sub-polar regions such as Canada. In Spain, hemp was used as a material for biodegradable face masks, while in tropical countries, e.g., Vietnam, the development of biodegradable face masks made from coffee beans has been also proposed. Bioplastics made from poly(hydroxy alkanoates) (PHA) and poly(hydroxy butyrate) (PHB) were also possible to use as biodegradable face masks. Yet, the virus filtration efficiency of these biodegradable face masks should be investigated further. The use of antiviral properties made from natural products such as neem oil, turmeric, basil, carom seed, or moringa seed, might be incorporated in making the biodegradable face masks [53].

## 5. Concluding Remarks

This study reviewed many potential approaches to advance the production of face masks via membrane technology. Membrane-based technology demonstrates an excellent filtration efficiency for the separation of micro-aerosol droplets and viruses. Facile approaches are available to further enhance the filtration efficiency of the current face mask materials. They can be done by maintaining the surface charge, hydrophobicity, antiviral-embedded nanoparticles, and pore-tuning methods. Furthermore, the virus adsorption onto the polymeric materials of the face mask is worth investigating to increase the filtration efficiency and virus ineffectuality. The approaches might become an option to overcome the shortage issue of face masks, although the cost efficiency must be calculated. Furthermore, the method should be practically feasible to be conducted by the community with a minimum requirement of technical knowledge.

## Figures and Tables

**Table 1:** Face masks filtration efficiency.

Face Masks	Penetration of Particles (%)	NIOSH NaCl Efficiency (%)	Reference(s)
Surgical Face Masks	44	54.72–88.4	[4,15]
N95 Respirator	0.01 (3M Vflex 9105 N95)<0.1 (3M 9320 N95)	98.15–99.68	[4,15]
Cloth Mask	95		[15]
